# How do fish miss? Attack strategies of threespine stickleback capturing non-evasive prey

**DOI:** 10.1242/jeb.247814

**Published:** 2024-11-14

**Authors:** Seth Shirazi, Timothy E. Higham

**Affiliations:** Department of Evolution, Ecology, and Organismal Biology, University of California, Riverside, Riverside, CA 92521, USA

**Keywords:** Accuracy, Swimming, Suction, Feeding, Prey capture, Predator–prey

## Abstract

Most predators rely on capturing prey for survival, yet failure is common. Failure is often attributed to prey evasion, but predator miscalculation and/or inaccuracy may also drive an unsuccessful event. We addressed the latter using threespine stickleback as predators and bloodworms (non-evasive) as prey. High-speed videography of the entire attack allowed us to determine the strike tactics leading to successful or missed strikes. We analyzed movements and morphological traits from 57 individuals. Our results reveal that kinematics drive the strike outcome and that failed strikes primarily arise from incorrect timing of mouth opening, often beginning too far from the prey for suction to be effective. This likely stems from the lack of integration between locomotion and feeding systems. Our study begins to unravel the important link between behavior and success in fish feeding.

## Teaser

**Summary:** Predatory fish often miss when trying to capture prey. Increased predator–prey distance was the primary driver of failed prey capture attempts in threespine stickleback.

## INTRODUCTION

Predators that capture prey for survival frequently miss the prey item. Across the diversity of vertebrates, success rates vary from less than 50% up to 85% in some predatory mammals, hover around 25% or less in some fish-eating birds, and range from 23% to 100% in fishes (Abrams, 1989; Vermeij, 1982). These rates are complex and may depend on both prey type and whether they were recorded under natural/semi-natural or laboratory conditions (Vermeij, 1982). Although many predators adopt an ambush or sit-and-wait strategy for capturing their prey (Benoit and Caruso, 2021; Jones and Whitford, 1989; Metcalfe et al., 1997; Nilsson et al., 2010; Sancho, 2000), many will fly, run or swim rapidly towards a mobile prey to capture and consume it. The latter involves both locomotion and feeding for successful capture, and the integration of these systems is, therefore, critical (Higham, 2007a). Ultimately, understanding the biomechanical bases of successful capture is critical for understanding patterns of evolution in predator–prey interactions (Higham et al., 2016).

A predator's capture success is influenced by multiple factors, including predator attack behavior and prey responses (e.g. Bhattacharyya et al., 2021; Lauder and Prendergast, 1992; Norton, 1991; Shifferman and Eilam, 2004), predator satiation levels (e.g. Sass and Motta, 2002), prey density (Combes et al., 2012), habitat structure (i.e. structural complexity; Crowder and Cooper, 1982) and environmental conditions [i.e. light intensity and turbidity (Benfield and Minello, 1996; Vinyard and O'Brien, 1976), temperature (Beddow et al., 1995) or hydrodynamic regime (China et al., 2017)]. Additionally, the morphology of the predator, such as body depth or structures contributing to suction, can also influence the ability to capture prey (Day et al., 2015; Rincón et al., 2007). Any shortcomings on either the predator attack or prey response may lead to an unsuccessful feeding attempt. Thus, capture failure can be a result of: (1) the prey avoiding capture (e.g. fleeing, detection avoidance, etc.), (2) flaws in the predator's approach or strike after the prey item has been detected (sometimes termed ‘intrinsic failures’; Nyberg, 1971) or (3) a combination of both. Intrinsic failures can be caused either by sub-optimal biomechanics (e.g. poor suction or locomotor speed) or poor sensorimotor integration (e.g. poor strike timing or trajectory), leading to reduced accuracy (Higham, 2007a; Martin et al., 2022; Milton and Bergmann, 2023; Montuelle and Kane, 2019; Rice and Westneat, 2005).

Prey capture in fishes can occur along a continuum from suction to ram feeding. Most fishes use some suction (Lauder, 1980; Wainwright et al., 2007), whereby predators rapidly expand their buccal cavity to generate a negative pressure inside the mouth relative to the surrounding fluid (Higham et al., 2006b; Muller et al., 1982; Van Leeuwen, 1984). This generates a flow of water into the mouth, entraining the prey in the ingested volume of water (Day et al., 2005). The predator must be very close to the prey (within one gape diameter) for suction to be effective (reviewed in Day et al., 2015). Consequently, suction feeders must execute well-timed strikes and precisely position their mouths near the prey (Higham et al., 2006a; Kane and Higham, 2014).

Predator attacks and prey evasion are often examined simultaneously (e.g. Jolles et al., 2022; Lucas et al., 2021; Webb, 1984; Webb and Skadsen, 1980), making it challenging to determine the specific biomechanical factors underlying successful attacks. To investigate the drivers of capture success, we examined the kinematics of predatory strikes of threespine stickleback (*Gasterosteus aculeatus*) attacking non-evasive prey. We hypothesized that flaws in the mechanical performance of the predator's attack, particularly variables related to suction-feeding and swimming performance, would be the main drivers of successful capture attempts. These variables include ram speed, time to peak gape (TTPG) and maximum gape (MG) (Higham et al., 2006a; Holzman et al., 2007; Sanford and Wainwright, 2002). Specifically, we predicted that successful strikes would exhibit a shorter TTPG, as a faster expansion of the mouth cavity results in more negative pressure (Higham et al., 2006b), leading to higher speeds of water entering the mouth, and therefore increased suction-induced drag experienced by the prey.

## MATERIALS AND METHODS

### Experimental subjects

Threespine stickleback (*Gasterosteus aculeatus* Linnaeus 1758) were collected around the Bamfield Marine Science Centre (BMSC; Bamfield, BC, Canada) under AUP RS-21(R19)-07. Fish were raised and bred in the lab until the F2 generation. Age and sex of each fish were not determined. Fish were fed a combination of blood worms and mysis shrimp. Fish were not fed the day before the feeding trials.

### Video collection

Feeding events were captured in lateral view using a Phantom Miro M110 high-speed camera (400 frames s^−1^). With the stickleback at one end of the filming tank (20 gallons, ≈76 l), dead bloodworms were introduced at the opposite end. If the predator did not attempt to strike from the farthest distance, additional prey items (one at a time) were dropped into the tank at closer distances to the fish until a strike was observed. Feeding trials continued (minimum of 2 min between trials) until the predator stopped feeding or appeared less aggressive, yielding us a range of trial numbers (1–9). Owing to the absence of 3D video tracking, we only analyzed trials where the strike was clearly perpendicular to the lens of the camera.

### Video analyses

Nine landmarks were digitized on the predator, prey and the background using DLTdv8 (Hedrick, 2008) in MATLAB 2022 (The MathWorks, Inc., USA) (Fig. S1). These points were subsequently used to calculate several kinematic variables (Table S1) using custom MATLAB code.

We analyzed 246 feeding sequences from 57 individuals, encompassing both successful and failed strikes. A successful strike was defined as capturing any part of the prey on the first attempt, while a failed strike was defined as missing the prey completely on the first attempt. Onset of slow mouth openings were excluded from analyses (see supplementary Materials and Methods).

### Morphometrics

Following the feeding trials, fish were euthanized with an overdose of Eugenol (clove oil). The fish was immediately placed on a piece of white plastic with a ruler in view. Photographs were taken and the images were analyzed using ImageJ (US National Institutes of Health, Bethesda, MD, USA). The right pectoral fin of each fish was removed at the body and photographed (fully extended) under a stereo microscope (Nikon SMZ800). Measurements included standard length, height, eye diameter, pectoral and caudal fin areas, pectoral fin length and ray count. Standard length and height were chosen to delineate variations in body size and depth, pectoral and caudal fin measurements were chosen owing to their impact on locomotor behavior such as positioning, braking or turning (Higham, 2007a,b; Higham et al., 2005; Rice and Hale, 2010), and eye (lens) diameter was included as it significantly correlates with visual acuity (Caves et al., 2017).

### Statistics

**Fig. 1.**
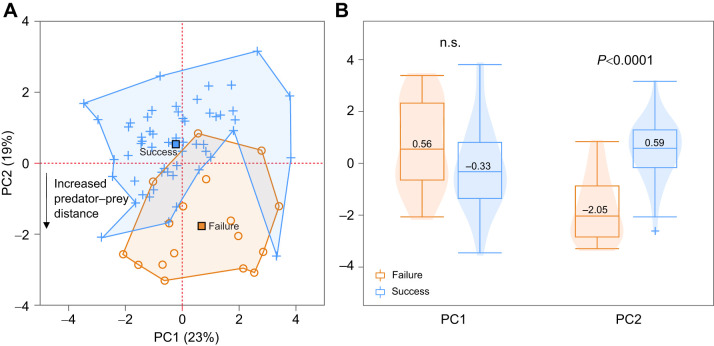
**Principal component analysis (PCA) results.** (A) Principal components 1 and 2 for kinematics. Successful strikes (*n*=56) are shown as blue plus signs and failed strikes (*n*=17) as orange circles. Filled squares indicate the average for the successful and failed strikes. The lines surrounding the clouds of points are drawn for visualization purposes only. (B) Welch's *t*-test (two-tailed) comparing PC scores (median values indicated) for successful and failed strikes. Kinematics along PC2 (*P*<0.0001) is the only significant driver of success/failure. Loadings are shown in Table S1: maximum gape (MG), ram speed at MG and distance traveled load positively on PC1; predator–prey distance (PPD) at strike initiation and PPD at MG load negatively on PC2.

We conducted principal component analyses (PCA) to reduce the dimensionality of the data and compare the kinematic variables and morphological traits between successful and failed strikes (Fig. 1A). To simplify the analysis and capture the most variation, only the first two PCs were reported for both morphology and kinematics. However, to ensure that PC3 onwards were not contributing to success/failure, we conducted logistic regressions comparing these PCs with success/failure and found no significant correlation. Restricted estimated maximum likelihood (REML) imputed data accounted for the very few kinematic columns that had missing values [13/962 values (0.01%)]. Statistical analyses were done using JMP Pro (version 16.2, SAS Institute, Cary, NC, USA). Kolmogorov–Smirnov tests confirmed the normality of our data prior to conducting parametric tests.

Because we were mainly interested in maximum performance, and owing to the fact that we had unequal sample sizes per individual, we used the kinematic variables of the maximum performing trial of each individual for all of the analyses. This was the trial with the shortest TTPG. If two trials had the same TTPG, we selected the trial with the largest MG, as this is an additional measure of suction performance among fishes (Higham et al., 2006a; Sanford and Wainwright, 2002; Holzman et al., 2007). If there was only one trial for success/failure, we used that as the maximum performance trial. There were only six individuals with one trial.

To determine whether body and/or fin morphology influenced capture success, we compared morphological features (using a PCA) of individuals with at least one failure with individuals who never missed. Morphology PC1 and PC2 scores (Table S1) were included as covariates in an analysis of covariance (ANCOVA) that included kinematic PC1 and PC2 scores, with success/failure as the categorial independent variable (Table S2). A full factorial approach was used to assess all interaction effects between covariates and success/failures.

For kinematics measurements, we compared the component values of PC1 and PC2 between successful/failed strikes using Welch's two-sample (two-tailed) *t*-tests (Fig. 1B). Welch's was chosen to account for the unequal sample size (Kim, 2019) between failed and successful strikes.

**Fig. 2.**
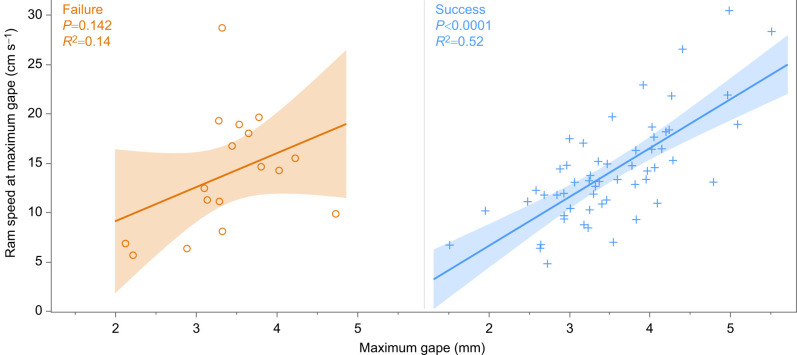
**The integration of feeding (MG) and locomotion (ram speed at MG) for 17 failed (left, orange) and 56 successful (right, blue) strikes.** Only successful strikes exhibited a significant correlation (*P*<0.0001). The shaded colored areas represent the 95% confidence intervals.

To examine differences in the integration of feeding and locomotion between successful and unsuccessful strikes, we performed linear regression analyses between MG and ram speed at MG (Fig. 2).

## RESULTS AND DISCUSSION

Premature opening of the mouth during the strike is the primary driver of failed strikes in threespine stickleback. Our multivariate analyses indicated that PC2 scores clearly differentiated failed and successful strikes (Fig. 1A), and the variables with the highest loadings on PC2 were predator–prey distance (PPD) variables (Table S1). PPD, the distance at which the strike is initiated, was significantly greater for failed strikes (mean: 3.88 mm) compared with successful strikes (mean: 1.53 mm; Fig. 1; Table S2). Our prediction that TTPG and ram speed would primarily impact the outcome of the strikes was not supported. In addition, PPD was not influenced by the distance that the predators traveled before strike initiation (Table S1), indicating that swimming from relatively long (e.g. >25 cm) or short (e.g. <2 cm) distances did not affect the distance at which the strike was initiated. This implies that errors involving sensorimotor integration likely underlie the inability to correctly time buccal cavity expansion needed to entrain the prey in the suction-generated flow field.

### What drives failure?

During suction feeding, peak fluid speeds are limited to a very short distance from the mouth aperture (Day et al., 2005, 2015; Higham et al., 2006a,b). Thus, generating peak suction when the prey is not within the flow field minimizes the ability to draw prey into the mouth. We found that the average PPD at MG was 0.31 mm for the successful strikes and 2.2 mm for the failed strikes (Fig. 1; Table S2). If we assume that stickleback suction is hydrodynamically similar to other species, this will result in ∼70% and ∼13% of maximum fluid speed at the location of the prey, respectively. Given that our study only used non-evasive prey, we expect failure rates to be even greater when feeding on evasive prey given the added possibility of a rapid escape maneuver executed by the prey.

### How do stickleback compare with other fishes?

Predatory errors, arising from decreased integration of locomotion and feeding, have been noted to influence capture success in multiple bony fish predators. Decreased strike accuracy via inadequate PPD is one of these factors and was important in our study. Other species exhibit the same pattern, including smoothhead and scalyhead sculpins attempting to feed on non-evasive crabs (Norton, 1991), northern pike feeding on golden shiners (Jolles et al., 2022), Hawaiian sleepers feeding on free swimming gobies (Maie et al., 2014), red lionfish feeding on live damselfish (Peterson and McHenry, 2022) and a recent non-fish example of praying mantises feeding on mealworms (Oufiero et al., 2024). When attacking prey with no evasive movements, largemouth bass can have an ineffective combination between ram speed and PPD (Nyberg, 1971). Specifically, bass are sometimes too slow at the time of mouth opening, which means they do not go far enough to engulf the prey (Nyberg, 1971). Similar velocity–PPD relationships are observed in chain pickerel (Rand and Lauder, 1981) and pike cichlids (Walker et al., 2005), although these studies involved evasive prey. Sensorimotor coordination errors are thought to drive strike failure in common snook when the prey does not initiate evasive movements (Caldentey et al., 2021). These examples include representatives across many feeding modalities, such as suction, ram and biting. This suggests that the underlying factors that drive feeding and locomotor integration, and strike success, may be similar across all feeding modes.

### Strike accuracy, morphology and capture success

Strike accuracy is the correct positioning of the mouth relative to the predator, and this can occur in one or more of the three axes (Higham et al., 2006a). For instance, accuracy can decrease owing to a misalignment of the strike trajectory relative to the location of the prey (lateral or vertical inaccuracy; e.g. Drost, 1987), as well as along the fore–aft axis (e.g. Nyberg, 1971), resulting in opening the mouth too early or too late. Research correlating aiming inaccuracies and capture success have found no convergence on a single axis of accuracy (Drost, 1987; Hawkins et al., 2023; Kane and Higham, 2014; Nyberg, 1971; Webb and Skadsen, 1980). Although we were not able to quantify accuracy along all three axes, future work that utilizes three-dimensional videography and a symmetrically shaped prey item will be able to determine accuracy when capturing both evasive and non-evasive prey. Using live prey will also determine to what extent prey movements can alter the predator's strike accuracy.

Despite the large variation in morphological traits among stickleback (see morphological loadings on Table S1), it is inconclusive whether morphology influences success. For kinematic PC2 scores, the sole axis of divergence between failed and successful strikes, only morphology PC2 (18% variation explained) was a significant covariate (*P*<0.01; Table S2). The variables with the strongest loading on PC2 were pectoral fin length and area (Table S1), suggesting that larger pectoral fins may increase the chance of failure. Future studies could assess the influence of morphology on kinematic variables during prey capture by including a much larger sample size and additional external and internal morphological traits.

### Importance of individual variation

Our study included almost 60 individuals of the same species, by far the most of any kinematic study of capture success in fishes. Variation was considerable (Table S2), with some individuals failing more often than others. Among five individuals of redbreast sunfish, Hawkins et al. (2023) uncovered a considerable amount of individual variation in feeding and locomotor kinematics during prey capture. For example, they found that PPD at strike initiation ranged from 0.42 to 3.09 cm. They generally found that locomotor traits were more variable than feeding traits, but it is unclear if this is a general pattern among fishes. Kane and Higham (2020) also found that the integration between locomotion and feeding during prey capture varied among individual bluegill sunfish. Future studies should aim to include many individuals to capture variation when feeding on non-evasive and evasive prey.

### Integration of locomotion and feeding

Having exceptional mechanical performance alone is unlikely to result in successful prey capture in mobile predators. For instance, an individual may generate very strong suction but still fail owing to incorrect timing of mouth opening (either too early or too late). Thus, it is the functional synergy between these systems that enables organisms to effectively hunt. Variables often correlated in fishes are ram speed at MG (locomotion) and MG (feeding) (reviewed in Kane and Higham, 2015). The general relationship is that fishes with greater attack velocities have larger gapes (Higham, 2007a; Oufiero et al., 2012). This is likely because swimming faster will decrease the time to accurately position the mouth close to prey to entrain it in the flow field generated by suction (Higham et al., 2006a). Therefore, having a larger gape will reduce the need to be as accurate given that the ingested volume of water is greater and the ‘reach’ of this volume extends both higher and farther in front of the predator (Higham et al., 2006a).

**Fig. 3.**
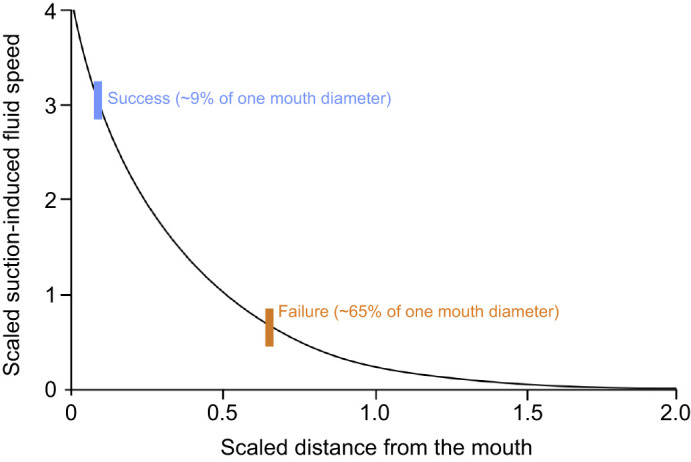
**The relationship between suction-induced fluid speed and distance from the mouth aperture.** Distances are proportionate to mouth diameters and flow speed equals 1 at half gape distance. This curve is redrawn from Day et al. (2015) and includes data from goldfish, largemouth bass, bluegill sunfish and modeling data. Superimposed on this plot are the mean values of successful and failed strikes from the threespine stickleback in our study.

In our study, the integration of MG and ram speed at the time of MG (represented as a linear regression; Fig. 2) was only significant for successful trials (success: *P*<0.0001, *R*^2^=0.52; failure: *P*>0.05, *R*^2^=0.14). This could explain why adequate PPD for effective suction is reached in successful strikes but not in failed strikes – precise timing of mouth expansion stems from the integration of gape size and ideal attack velocity in successful strikes. This is because the attack velocity during the pursuit can influence gape size (Kane and Higham, 2014; Higham, 2007a), and gape size determines maximum effective suction distance (Fig. 3).

### Future directions

Incorrect PPD occurs when the predator cannot accurately identify the position of the prey or when the predator mistimes the opening of its mouth. For instance, ablating certain neurons in the zebrafish brain increases strike failure by impeding the ability to locate prey when it is within the binocular strike zone, while exerting no influence on the initiation of the hunting sequence (Gebhardt et al., 2019; Zhu and Goodhill, 2023). This could explain why predators miscalculate the position of the prey, and subsequently strike prematurely. Perhaps some stickleback in our study had a poor ability to locate the prey, stemming from various sensory issues such as poor depth perception or low visual acuity. However, we found that eye size was not different between individuals that never missed (successful) and those who missed at least once (failed) (Table S2), and eye size is a correlate of visual acuity (Caves et al., 2017). Thus, visual acuity is likely not the issue that the unsuccessful stickleback faced in our study. Instead, the integration of sensory and motor information was potentially faulty, and could be explored in the future by recording neural activity in free-swimming fish during predator–prey interactions (Gibbs et al., 2023). Another way to determine what drives individuals to be more successful is by leveraging the existing stickleback genome (Reid et al., 2021) in order to identify candidate loci that are linked to sensory or locomotor traits.

Our study examined prey capture in a very controlled environment, with non-evasive prey and still water. How prey capture occurs in realistic situations, including evasive prey, flowing water, different temperatures and in turbid water, would yield a greater understanding of capture success (and failure) in nature (Higham et al., 2015). For example, sub-optimal temperatures could negatively impact the relationship between swimming and mouth opening given the influence that temperature has on visual acuity (Fritsches et al., 2005), muscle performance (Rome et al., 1990) and sensorimotor pathways (van den Burg et al., 2006).

## Supplementary Material

10.1242/jexbio.247814_sup1 Supplementary information

Table S2. Spreadsheet containing four sheets of results and analyses. First sheet (“Kinematics”) shows the kinematic data of maximum perform performing successful and/or failed trials of each individual. Refer to Table S1 for variables in unabbreviated states. Red text indicates Restricted Estimated Maximum Likelihood (REML) imputed data. “PC1” and “PC2” represent the component values derived from the kinematics PCA (Fig. 1). Individual 17 excluded in analyses analyses. Second sheet (“Morphology”) shows the morphological data of individuals with at least one failure to individuals who never missed. “PC1” and “PC2” represent the component values derived from the morphology PCA (Fig. 2). Individual 17 excluded in analyses. Third sheet (“Trials”) shows the total number of trials, including successful and failed strikes. Maximum performance trials represent the maximums for both successful and unsuccessful attempts. Approximately 90% of all trials were successful. Fourth sheet (“ANCOVA) shows the effect tests of the ANCOVAs. Kinematic PC scores were selected as dependent variables (A tests of the ANCOVAs. Kinematic PC scores were selected as the dependent variables (A represents PC1; B represents PC2). Success/Failure was the categorical independent variable, and morphological PC1 and PC2 scores were implemented as con tinuous covariates. * Represents the interaction effects.
