# Supplementary material for: How do fish miss? Attack strategies of threespine stickleback capturing non-evasive prey
Source: J Exp Biol. 2024 Nov 14;227(22):jeb247814. doi: 10.1242/jeb.247814 (PMC11586519; doi:10.1242/jeb.247814)
Supplement: Supplementary information [file jexbio-227-247814-s1.pdf]

SUPPLEMENTARY MATERIALS & METHODS

We excluded the slow onset of mouth opening stages from our analyses, and only in a few, the decision was not straight forward (<20 trials). This is because in suction-feeders, strike initiation is generally measured at onset of the fast mouth opening stage, usually quantified as the moment the mouth reaches 20% gape (see Sanford and Wainwright 2002, Day et al. 2005). 20% was not always reflective of the rapid mouth opening stage in sticklebacks, so we manually checked every trial to ensure accurate identification of the onset of rapid mouth opening.

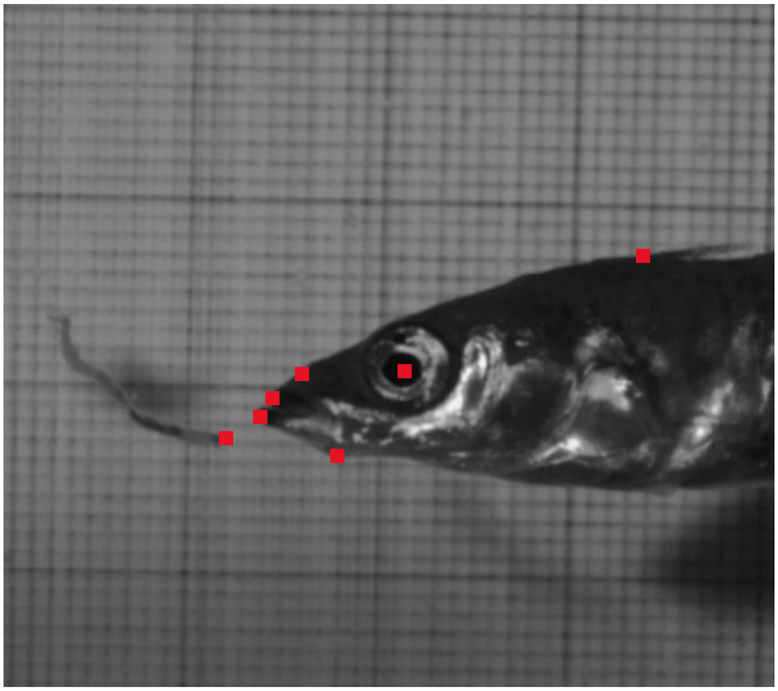

Fig S1. Landmarks digitized on the predator, prey and background using DLTdv8 (Hedrick, 2008) in MATLAB 2022 (The MathWorks, Inc., USA). Landmarks included the center of the eye, anterior end of the first dorsal spine, anterior end of the upper jaw, anterior end of the lower jaw, ventral-most point of the hyoid, a point near the upper jaw that is not protruded and a point indicating the edge closest to the fish's mouth. Two background landmarks on the left and right side of the grid were also digitized for scaling purposes (not shown here).

Journal of Experimental Biology • Supplementary information

Table S1. Loading matrix for the PCA of kinematic (A) and morphological (B) measurements. Noteworthy are bolded loadings with absolute values exceeding 0.6. Note that greater values of maximum deceleration are on the right-hand side, thus it appears that it is loading negatively. In reality, it should be considered as the opposite.

**A. Kinematics Variables**	**PC1**	**PC2**
Time to Peak Gape	-0.0415	-0.0859
Maximum Gape	**0.63481**	0.38866
Maximum Cranial Elevation	0.03771	0.04474
Maximum Jaw Protrusion	0.36275	0.45341
Maximum Hyoid Depression	-0.2767	0.5589
Timing of Hyoid Depression Relative to Maximum Gape	-0.3737	0.27789
Maximum Deceleration	**-0.6494**	-0.1532
Time of Maximum Deceleration Relative to Maximum Gape	-0.1041	0.3935
Ram Speed at Maximum Gape	**0.87105**	0.25383
Distance Traveled	**0.69232**	0.27099
Predator-Prey Distance at Strike Initiation	0.46958	**-0.7224**
Predator-Prey Distance at Maximum Gape	0.34407	**-0.8311**

**B. Morphology Variables**	**PC1**	**PC2**
Standard Length	**0.89843**	0.14999
Height	**0.82396**	-0.0004
Eye Diameter	**0.60931**	0.49962
Caudal Fin Area	**0.71313**	0.22032
Pectoral Fin Length	**0.64339**	**-0.654**
Pectoral Fin Area	**0.69213**	-0.5368
Ray Count	0.41433	0.47381

Journal of Experimental Biology • Supplementary information

Table S2. Spreadsheet containing four sheets of results and analyses. First sheet ("Kinematics") shows the kinematic data of maximum performing successful and/or failed trials of each individual. Refer to Table S1 for variables in unabbreviated states. Red text indicates Restricted Estimated Maximum Likelihood (REML) imputed data. "PC1" and "PC2" represent the component values derived from the kinematics PCA (Fig. 1). Individual 17 excluded in analyses. Second sheet ("Morphology") shows the morphological data of individuals with at least one failure to individuals who never missed. "PC1" and "PC2" represent the component values derived from the morphology PCA (Fig. 2). Individual 17 excluded in analyses. Third sheet ("Trials") shows the total number of trials, including successful and failed strikes. Maximum performance trials represent the maximums for both successful and unsuccessful attempts. Approximately 90% of all trials were successful. Fourth sheet ("ANCOVA) shows the effect tests of the ANCOVAs. Kinematic PC scores were selected as the dependent variables (A represents PC1; B represents PC2). Success/Failure was the categorical independent variable, and morphological PC1 and PC2 scores were implemented as continuous covariates. * Represents the interaction effects.

Available for download at
https://journals.biologists.com/jeb/article-lookup/doi/10.1242/jeb.247814#supplementary-data
